# Supplementary material for: Multi-domain translation between single-cell imaging and sequencing data using autoencoders
Source: Nat Commun. 2021 Jan 4;12:31. doi: 10.1038/s41467-020-20249-2 (PMC7782789; doi:10.1038/s41467-020-20249-2)
Supplement: Supplementary file 1 — Supplementary Information [file 41467_2020_20249_MOESM1_ESM.pdf]

Supplementary Information: Multi-Domain Translation between Single-Cell Chromatin Imaging and Sequencing Data using Autoencoders

Karren Dai Yang Anastasiya Belyaeva Saradha Venkatachalapathy
Karthik Damodaran Abigail Katcoff Adityanarayanan Radhakrishnan
G.V. Shivashankar Caroline Uhler

November 15, 2020

Supplementary Methods

Model validation on paired RNA-seq and ATAC-seq data

We obtained paired RNA-seq and ATAC-seq data collected in the same cell from [1]. Specifically, we used paired data collected from human lung adenocarcinoma–derived A549 cells treated with dexamethasone (DEX) for $0, 1$, or 3 hours. We downloaded the single-cell RNA-seq data from the GEO accession number GSE117089, corresponding to [1]. For the ATAC-seq data, instead of using raw matrix of peaks by cells, we acquired a transcription factor (TF) motif by cells matrix from the authors (Supplementary Data 1), which was computed as described in [1] by counting occurrences of each motif in all accessible sites for each cell, resulting in 815 TF motifs. For single-cell RNA-seq data we considered genes that were determined to be differentially expressed by [1], keeping genes with q-value > 0.05. Both single-cell RNA-seq and ATAC-seq were $\log(x+1)$ transformed and normalized to zero mean and unit variance. The number of cells that were shared between TFs $\times$ cells matrix from ATAC-seq and genes $\times$ cells matrix from RNA-seq was 1874, therefore our model had to learn a latent embedding and translate between data sets with different number of features, i.e. for single-cell RNA-seq a matrix of 2613 genes $\times$ 1874 cells and for single-cell ATAC-seq a matrix of 815 TFs $\times$ 1874 cells.

We trained our cross-modal autoencoder model to embed single-cell RNA-seq and ATAC-seq into the same latent space (dimensionality of 50), which allows mapping and translation of samples from one space to the other. Our model's architecture consisted of fully connected layers with input, hidden layers and output sizes listed in Supplementary Table 1. In order to train the model we minimized the weighted sum of losses listed in Supplementary Table 2. The model was trained in Pytorch with learning rate of 0.0001 and batch size of 32 for 4000 epochs using Adam with $\beta_1 = 0.5, \beta_2 = 0.999$ and weight decay of 0.0001.

Since the RNA-seq and ATAC-seq data was collected in the same cell, we could evaluate the accuracy of our method in matching samples from RNA-seq to ATAC-seq (and vice-versa). For evaluation, we created an 80-20 training-test split of the paired data. To measure the accuracy of matching RNA-seq and ATAC-seq samples in the latent space or in the original space for methods that do not rely on the latent space, we used the following k-nearest neighbors accuracy, calculated on the test set:

$$\text{k-NN}(\text{A}, \text{B}) = \frac{\sum_i 1(b'_i \in a^k_i)}{n}, \tag{1}$$

where n is the length of the test set, A and B are sets of vectors, with b_i as a vector in B and a_i as its pair in A, and b'_i and a'_i are the encoded versions of b_i and a_i in the latent space. The set a^k_i contains the k nearest neighbors of a'_i in A', the set of vectors in A projected into the latent space. Since $\text{k-NN}(\text{A}, \text{B})$ does not necessarily equal $\text{k-NN}(\text{B}, \text{A})$, we computed the average of these metrics. We used ℓ_1 distance for distance computations in the latent space.

In order to quantify whether our model maps cells to the correct treatment time cluster, we computed the fraction of cells in the test split that had the correct cluster assignment. In order to assign cells to a cluster, we trained a simple logistic regression classifier on the latent space using cells in the training split and their corresponding treatment time labels. Subsequently, the trained classifier was used to predict treatment time labels on the cells in the test set and the accuracy of the classifier was quantified.

First, we compared our method against deep canonical correlation analysis (DCCA), which uses paired samples between two domains to learn a shared embedding of the two domains by maximizing the total correlation [2]. The model for DCCA consisted of two neural networks, one for each domain. For ATAC-seq data, the input to the model was a matrix with 815 features, followed by 815 hidden nodes with sigmoid activation, and a final output layer of size 50. For RNA-seq data, the input to the model was a matrix with 2613 features, followed by 2613 hidden nodes with sigmoid activation, and a final output layer of size 50. Finally, as in [2], linear CCA was applied to the output layers of the two neural networks corresponding to the two different domains. DCCA jointly learns the parameters for both neural networks such that the correlation of the final output layer between the domains is maximized. DCCA was trained using RMSProp with learning rate of 10^{-3}, batch size of 1024 for 100 epochs. Regularization parameter of 10^{-9} was applied to the networks.

For both our cross-modal autoencoder method and DCCA, we explored the use of samples whose pairing is known between the two domains (i.e., anchored cells in both datasets), which is available in some applications. To make use of the pairing information in our cross-modal autoencoder model, we included an additional term in the loss function corresponding to the mean absolute error between the paired training points in the latent space. While our method based on autoencoders does not require paired samples, DCCA does. In order to train DCCA with 0% paired samples, we randomly generated

paired samples using the treatment time labels of the cells as follows. For each point with a particular treatment time label, we sampled 100 random points with the same label to use as its paired samples.

We additionally compared our method against a popular method for data integration, Seurat version 3.0 [3, 4]. Briefly, this method assumes that the features across different modalities are the same and learns a shared embedding using CCA based on this assumption. In order to apply Seurat to this particular dataset, we used the Seurat pipeline as follows: in order to obtain from ATAC-seq data a matrix that has the same features as the gene expression matrix, the ATAC-seq data was transformed into a gene activity matrix using the CreateGeneActivityMatrix function in Seurat 3.0. We normalized and scaled the data using the NormalizeData and ScaleData functions in Seurat 3.0. Finally, a shared CCA embedding was learned using the FindTransferAnchors functionality in Seurat 3.0. Similar to our cross-modal autoencoder and DCCA, we used the inferred CCA embedding to quantify the method's performance. Note that Seurat was fit using both training and test data, thereby giving Seurat an advantage over the other methods.

Finally, we compared our method against CycleGAN [5], a prominent deep learning method for domain translation, which ensures that source samples are recovered back after mapping source samples to target domain and back to the source domain. We used the code provided by the authors of CycleGAN at `http://github.com/junyanz/pytorch-CycleGAN-and-pix2pix` to translate ATAC-seq to RNA-seq and RNA-seq to ATAC-seq. We modified the architecture of the generator and discriminator networks to handle non-image data and match the architecture of our cross-modal autoencoder. In particular, the generator for translating ATAC-seq to RNA-seq consisted of a sequence of fully-connected layers with the following sizes: 815, 815, 815, 100, 50, 100, 2613, 2613, 2613. Similarly, the generator for translating RNA-seq to ATAC-seq consisted of a sequence of fully-connected layers with the following sizes: 2613, 2613, 2613 815, 815, 815, 100, 50, 100, 815, 815, 815. The discriminator model for ATAC-seq data took as input 815 features, followed by 815 hidden nodes and then 100 hidden nodes with a final output layer of size 1. The discriminator model for RNA-seq data took as input 2613 features, followed by 2613 hidden nodes and then 100 hidden nodes with a final output layer of size 1. All models used leaky ReLU as activation. The CycleGAN was trained for 2000 epochs with a learning rate of 0.0002 and batch size of 32. We evaluated the model only in terms of the k-nearest neighbor accuracy since the fraction of cells in the correct cluster was meant to evaluate the quality of the latent space. The k-nearest neighbor accuracy of the CycleGAN was computed in the original instead of the latent space since the model does not rely on the latent space for domain translation. Similarly, we compared our method against MAGAN [6], which has an additional correspondence loss term that ensures the measurements coming from the same sample should be close to each other. We trained MAGAN by providing 5%, 50% and 100% of paired samples in the training data for the correspondence loss.

Gene expression data of naive CD4+ T-cells

We used gene expression data corresponding to human peripheral blood mononuclear cells (PBMCs) collected in [7]; the filtered cell by gene matrix was downloaded from `https://support.10xgenomics.com/single-cell-gene-expression/datasets/2.1.0/pbmc8k`. We analyzed the PBMC 8k data set since it had the highest number of reads per cell. Since the data was already filtered, we only applied minor additional filtering such as removing cells with high proportion of counts in mitochondrial genes ($\geq 10\%$), which reduced the number of cells from 8381 to 8371 cells. After cell filtering, we performed gene filtering by removing mitochondrial genes and keeping genes which had at least 10 cells expressing the gene with a count > 1, resulting in 7633 remaining genes.

After cell and gene filtering, we followed a standard analysis pipeline using Seurat (version 2.3.0) [3, 4]. We normalized the gene expression measurements for each cell by the total expression for that cell and scaled the result using the median total expression across cells. The scaled result was $\log(x + 1)$ transformed. We z-scored the data and applied PCA to obtain 30 components, which were used for t-SNE and clustering analysis. The t-SNE embedding for all cells, computed using default parameters, is shown in Supplementary Fig. 1a. We clustered the data using default clustering in Seurat (FindClusters function in Seurat version 2.3.0) with resolution parameter of 0.4, which resulted in 13 clusters, shown in Supplementary Fig. 1a. Briefly, the clustering method in Seurat constructs a k-nearest neighbor graph and adjusts the edge weights between cells based on Jaccard similarity of their local neighborhoods. The resulting graph is clustered using the Louvain algorithm to obtain cell clusters. In order to determine the identity of each cluster we performed differential expression analysis using the default Wilcoxon rank sum test in Seurat (FindAllMarkers function in Seurat version 2.3.0). We list the top 10 differentially expressed genes for each cluster in Supplementary Table 3.

From the clustering analysis of all PBMCs and annotation using differentially expressed genes, we were able to determine that cluster 1 generally corresponds to naive CD4+ T-cells (differential overexpression of CCR7, LEF1, TCF7), cluster 2 corresponds to cytotoxic T-cells (differential overexpression of GZMK, NKG7, CCL5), cluster 3 corresponds to activated CD4+ T-cells (differential overexpression of IL7R, IL32) and cluster 4 corresponds to naive CD8+ T-cells (differential overexpression of CD8A, CD8B, LEF1, CCR7) [8, 9]. Supplementary Fig. 1b provides a t-SNE plot of all PBMCs, colored by the expression of known markers genes, further corroborating our cell type annotation.

Autoencoder training on chromatin images for validation

We trained a convolutional autoencoder with the following architecture on the chromatin images: (1) We used 15 convolutional layers with 256 3×3 filters per layer followed by leaky ReLU activations throughout; (2) layers 2-6 have a stride size of 2 and layers 8-12 are followed by bilinear upsampling layers with a scale factor of 2. The bottleneck of our network thus provides a 256 dimensional representation of the images. We trained our network using the Adam optimizer (learning rate of 10^{-4}) and used a Kaiming uniform initialization for all our convolutional layers. All of the images were trimmed to remove background and resized to 32×32 images in order to remove nucleus size as a distinguishing feature. We held out 10% of the data as test data and trained until the reconstruction loss on the test data was smaller than 10^{-3}.

In order to determine whether our network was able to separate the poised and quiescent naive CD4+ T-cell clusters (as determined by the protein ratio of CORO1A to RPL10A) in an unsupervised fashion, we visualized the embedding of the images corresponding to the histogram peaks in Fig. 5c (namely the images with protein ratio in the range [.64, .7] and [0.93, 1]). Supplementary Fig. 7 shows the resulting t-SNE embedding, where the color coding corresponds to the protein ratio of CORO1A to RPL10A. Interestingly, the latent embedding of the images obtained in an unsupervised fashion (with no information about the proteins) captures the protein ratio.

Supplementary Figures

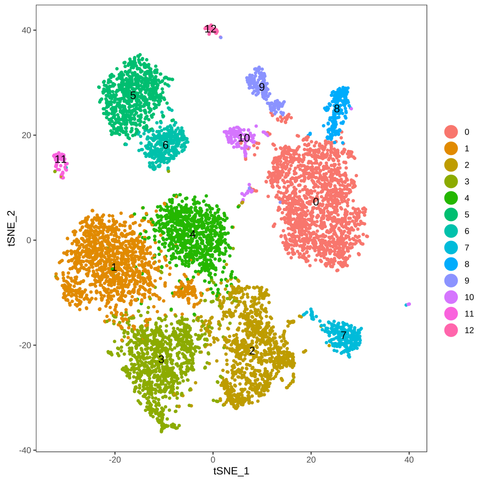

(a)

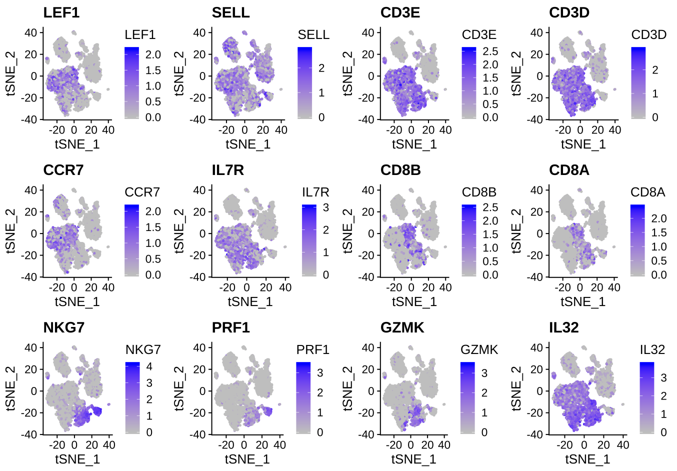

(b)

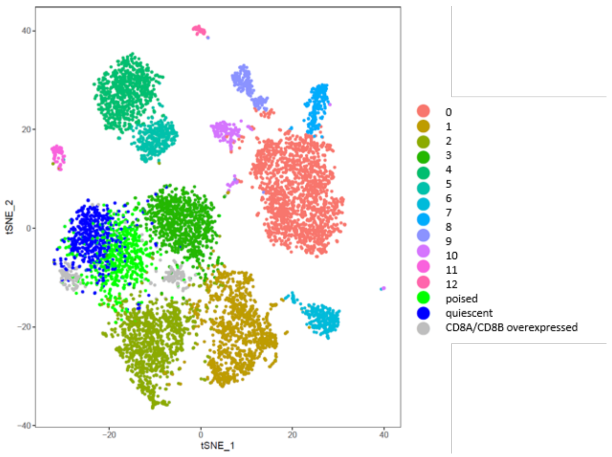

(c)

Supplementary Fig. 1: Clustering of peripheral blood mononuclear cell (PBMC) data. (a) t-SNE of all cells in the PBMC data set, colored by inferred cluster label. (b) t-SNE plots of all cells in the PBMC data set, colored by expression of genes marking naive T-cell subpopulations (LEF1, SELL, CCR7), T-cells (CD3E, CD3D, IL7R, IL32), CD8 T-cells (CD8A, CD8B), natural killer, and cytotoxic T-cells (NKG7, PRF1, GZMK). (c) t-SNE plot including the clustering of the naive CD4+ T-cells (clusters denoted by different colors). Grey subpopulation differentially overexpresses CD8A and CD8B as the genes with highest average log-fold change (corrected p-value $= 1.30 \times 10^{-40}$ and 1.54×10^{-51} respectively), indicating that these cells are not naive CD4+ T-cells; thus they have been removed from further analysis.

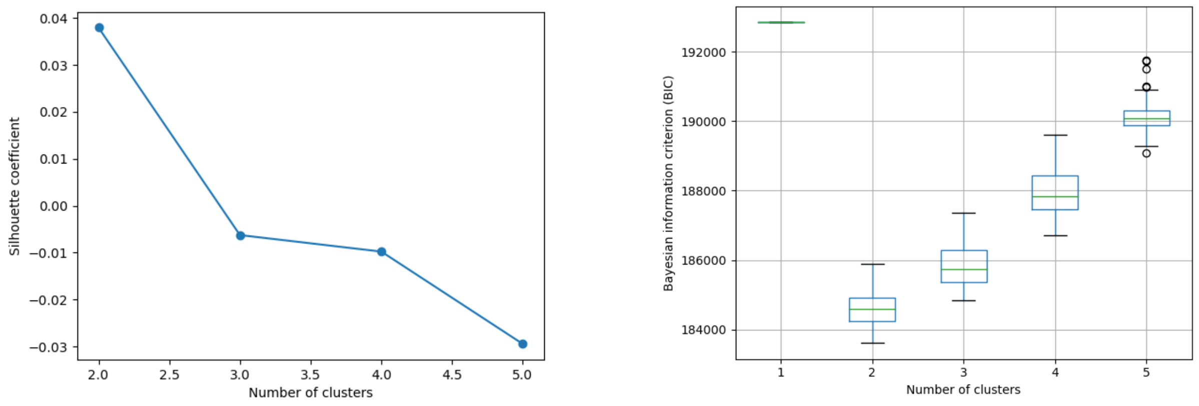

(a) (b)

Supplementary Fig. 2: Evaluating optimal number of clusters for naive CD4+ T-cell gene expression data ($n = 1166$ cells). (a) Silhouette coefficient for clusters obtained with Seurat at different resolutions (0.8, 0,9, 1.1, 1.15). (b) BIC score (averaged over 100 trials) for Gaussian mixture model with 1, 2, 3, 4 and 5 components. The boxplots illustrate the median (middle line), with box indicating the first and third quartiles and the whiskers indicating $\pm$ 1.5 $\times$ interquartile range. Outliers are plotted as separate dots.

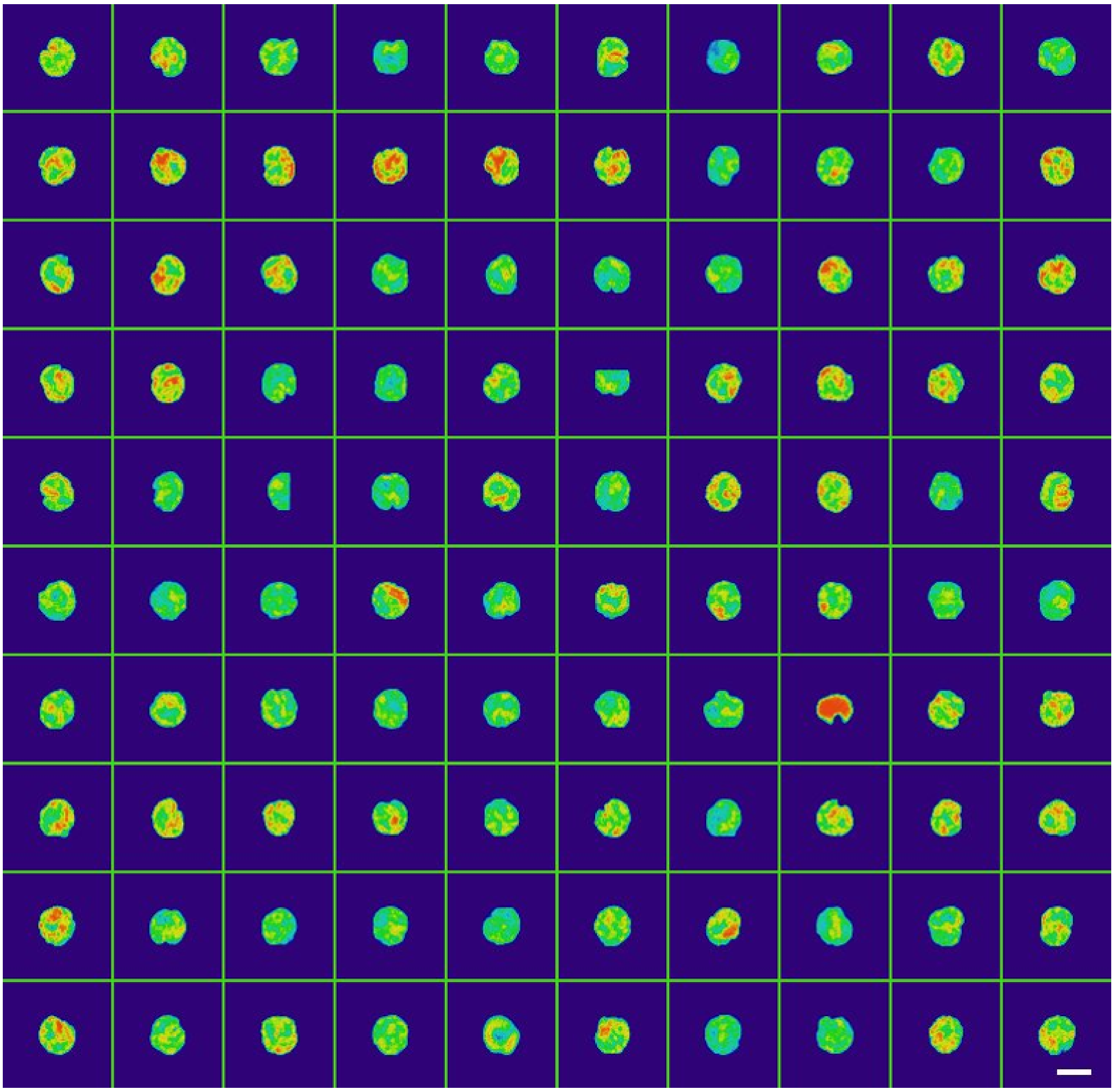

Supplementary Fig. 3: Examples of naive CD4+ T-cell nuclei stained with DAPI. Scale bar is 2 microns. Images were selected randomly from 4 experiments.

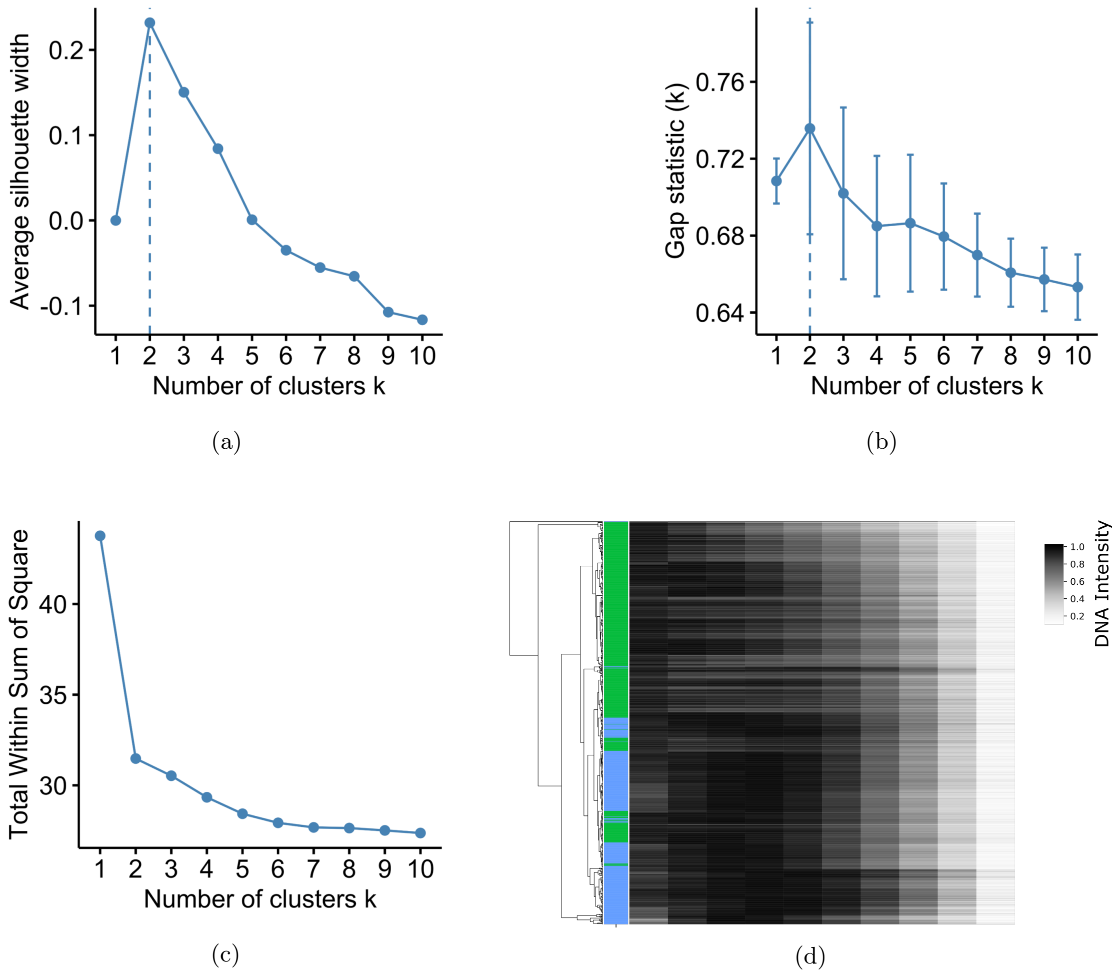

Supplementary Fig. 4: Evaluating optimal number of clusters for T-cell imaging data ($n = 729$ cells from two biologically independent replicates) using (a) average silhouette width, (b) gap statistic using 50 bootstrap samples, (c) total within-cluster sum of square, (d) alternative clustering using 1 - Pearson's correlation matrix with average linkage. Green and blue colors represent labels obtained based on the original clustering with 1 - Spearman's correlation and complete linkage, where green indicates the subpopulation of cells with central chromatin pattern and blue indicates the subpopulation of cells with peripheral chromatin pattern.

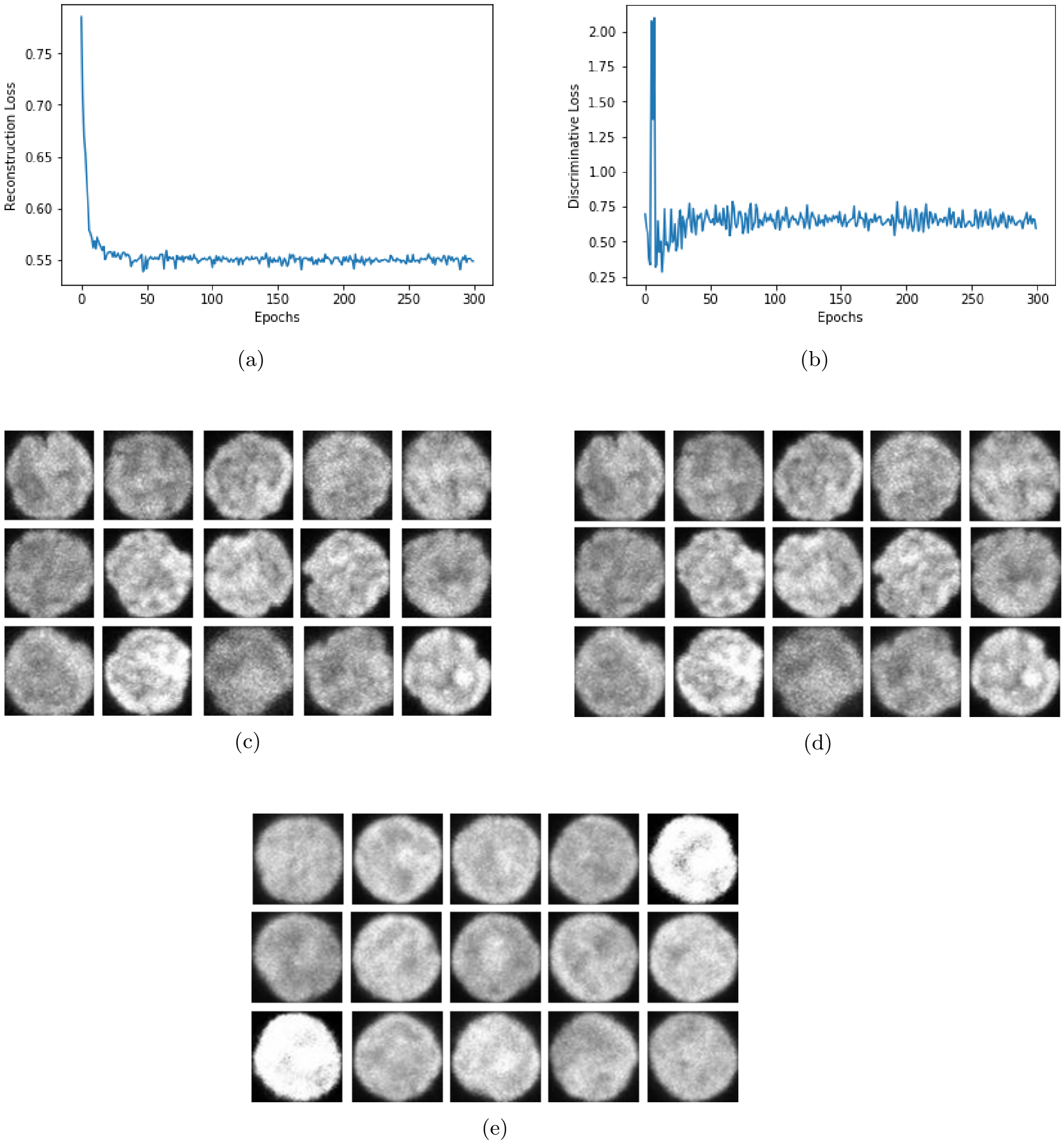

Supplementary Fig. 5: Model trained on single-cell RNA-seq and single-cell images of DAPI-stained nuclei. (a) Reconstruction loss curve (sum of RNA-seq and image reconstruction losses). (b) Discriminative loss curve for RNA-seq and image translation model. (c) Examples of input images to the image autoencoder. (d) Reconstructed images after training the image autoencoder. (e) Generated images translated from RNA-seq to image space. Images were selected randomly.

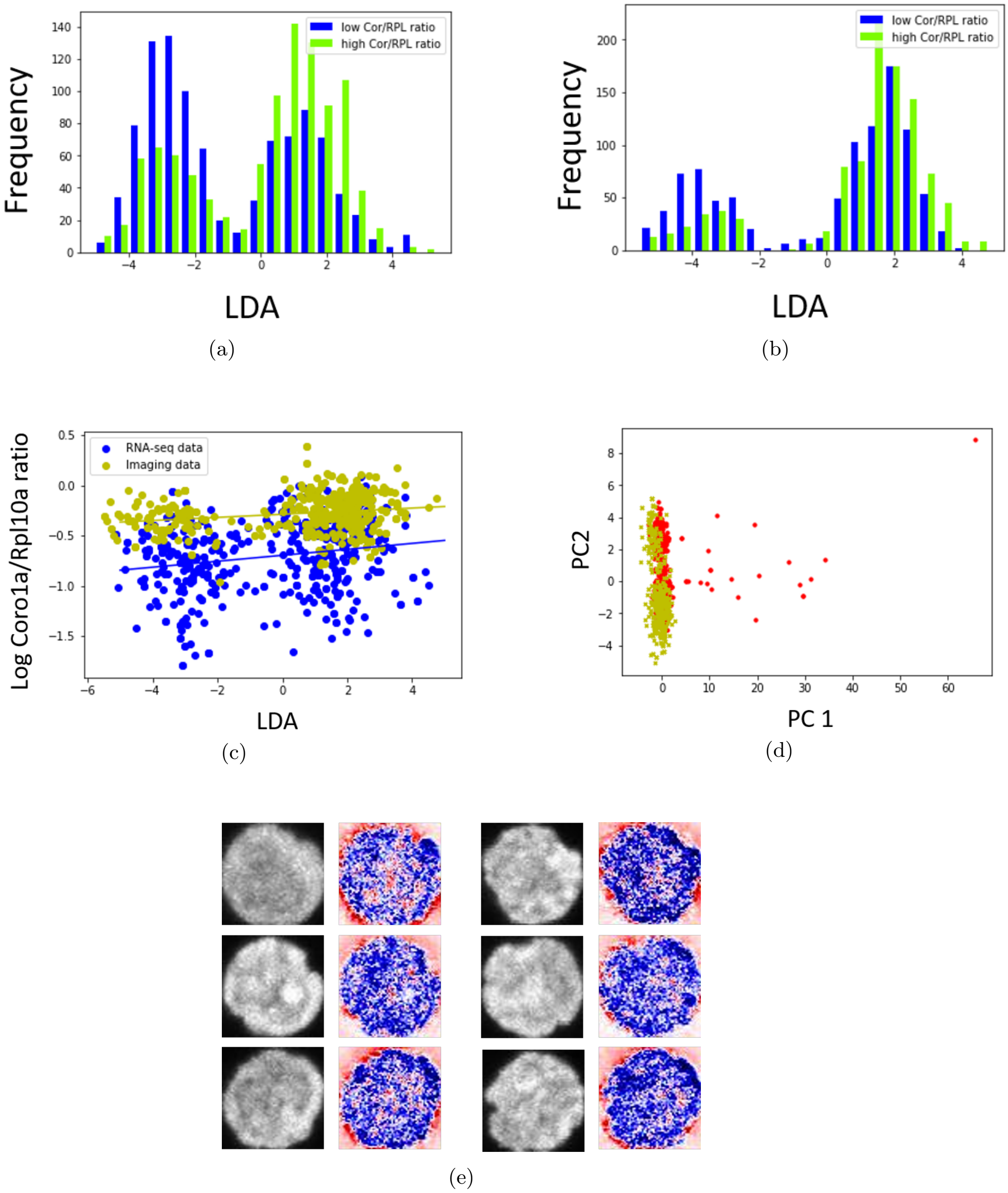

Supplementary Fig. 6: Validation of inferred latent embedding. Histograms of embedded naive CD4+ T-cells from (a) RNA-seq and (b) imaging data sets, split by high (green) versus low (blue) CORO1A/RPL10A ratio. Histogram is computed along LDA axis that maximally separates two subpopulations in the latent space, showing that the axis aligns with CORO1A/RPL10A ratio. (c) Scatterplot of CORO1A/RPL10A ratio versus projection onto LDA axis. In both data sets, RNA-seq (blue) and imaging (yellow), the positive correlation between the ratio and the projection onto the LDA axis is statistically significant ($p = 8.27 \times 10^{-6}$ for RNA-seq data, $p = 2.27 \times 10^{-6}$ for imaging data, two-sided Wald test for linear fit). (d) RNA-seq (red) and imaging (yellow) data embedded in latent space, visualized using PCA. (e) Interpretation of image features along the LDA axis that maximally separates the two naive T-cell subpopulations in the latent space. Results show decreased background chromatin concentration in the nucleus.

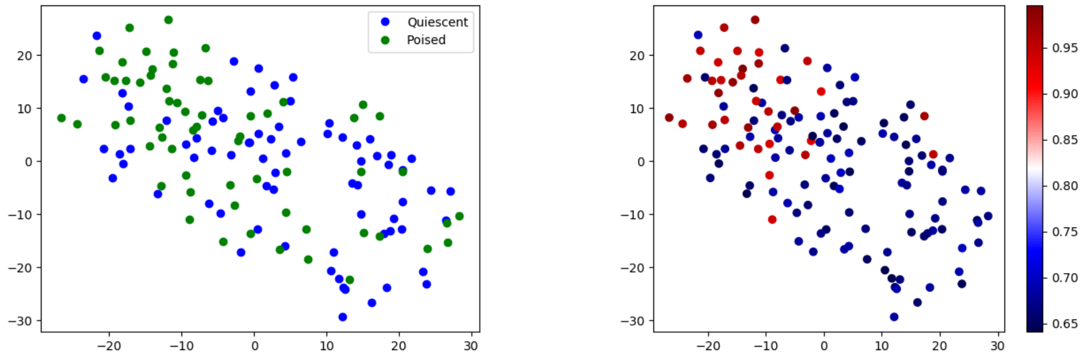

(a) (b)

Supplementary Fig. 7: t-SNE visualization of latent space for convolutional autoencoder colored by (a) cluster label: quiescent (blue) and poised (green) naive CD4+ T-cells and (b) protein ratio of CORO1A to RPL10A. The autoencoder separates out the two naive CD4+ T-cell clusters by protein ratio without cluster supervision.

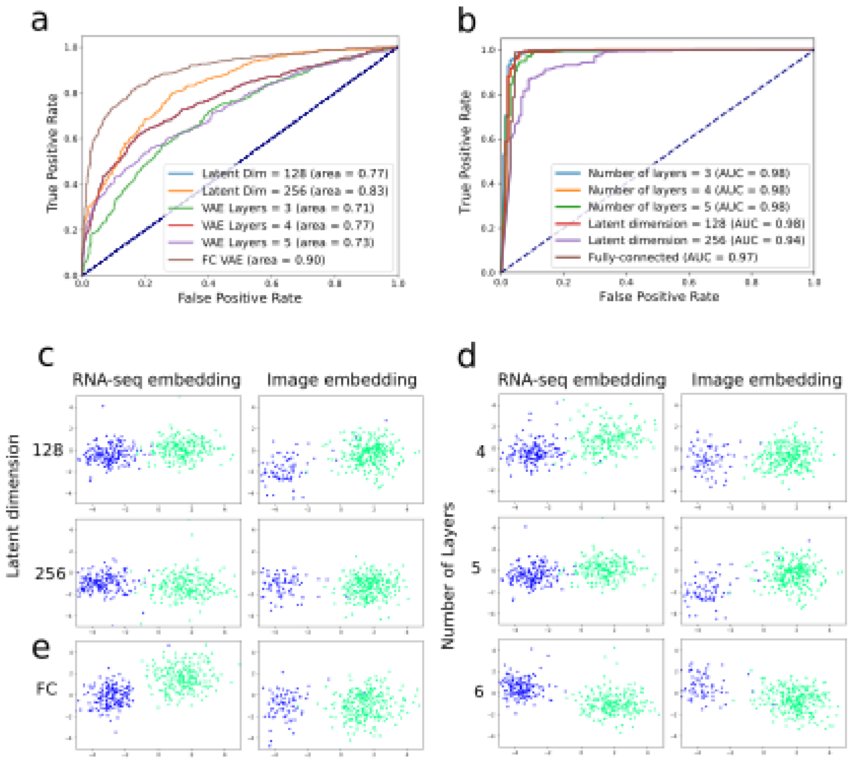

Supplementary Fig. 8: Evaluation of robustness to the choice of architecture (fully-connected versus convolutional layers, number of layers, and latent space dimension) for the cross-modal autoencoder integrating RNA-seq and chromatin imaging. (a) Receiver Operating Characteristic (ROC) curve illustrating performance of classifiers trained to distinguish between peripheral and central chromatin patterns in images when evaluated on images translated from RNA-seq data. High performance of classifiers indicates that the alignment of the clusters in the latent space also holds in the original gene expression and imaging spaces and is robust to different architecture choices. The dotted dark blue line represents random guessing based on evenly-distributed classes and the remaining colors represent different model architectures. (b) ROC curves illustrating performance of classifiers trained to distinguish between quiescent and poised gene expression programs when evaluated on RNA-seq data translated from images. (c) Linear Discriminant Analysis (LDA) plots of single-cell RNA-seq (left) and imaging (right) datasets embedded in the latent space for models with different numbers of latent dimensions. The clusters with more quiescent (blue) and poised (green) gene expression programs from the RNA-seq dataset are aligned with the clusters with peripheral (blue) and central (green) chromatin patterns from the imaging dataset. (d) Same as (c), for models with different numbers of layers in the RNA-seq VAE. (e) Same as (c), for model with fully-connected image VAE. Note that the model with latent dimension of 128 is the same model as the one with 4 layers in the RNA-seq VAE.

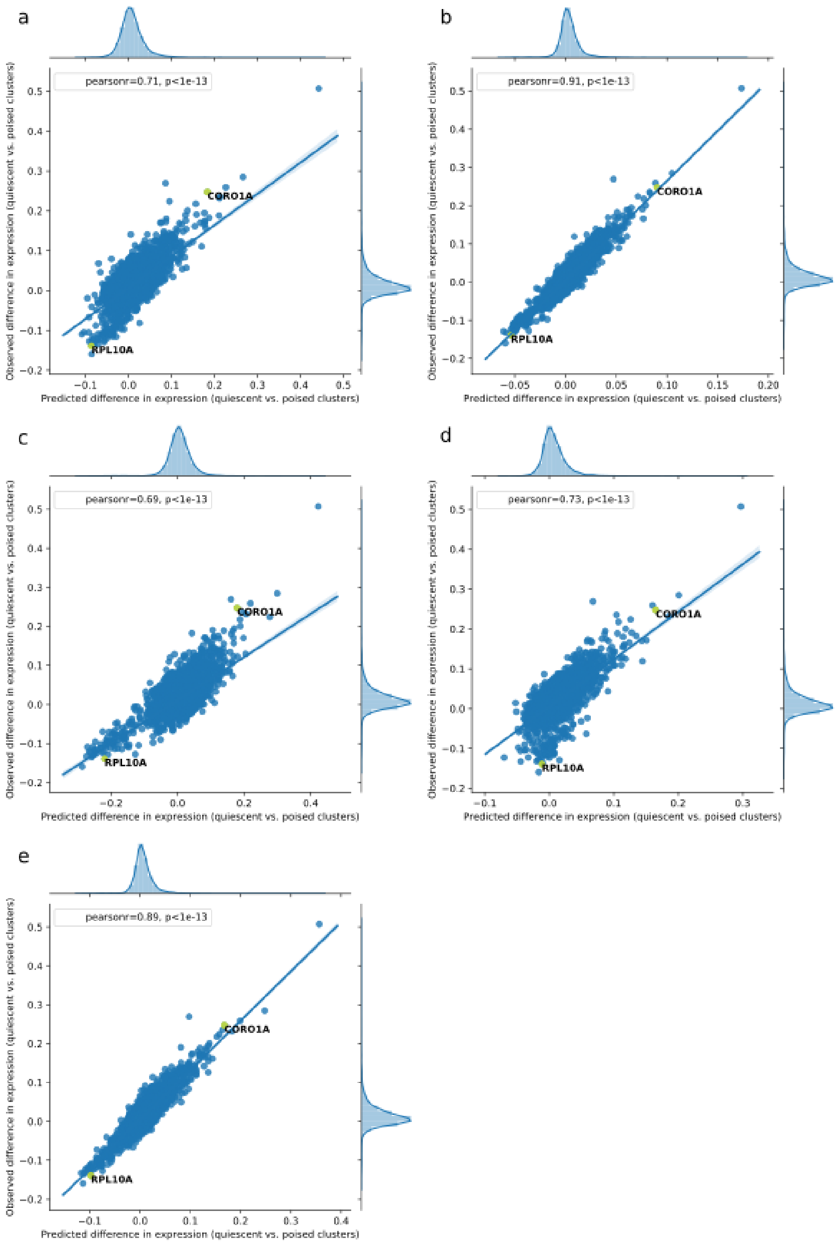

Supplementary Fig. 9: Evaluation of robustness to the choice of architecture (fully-connected versus convolutional layers, number of layers, and latent space dimension) for the cross-modal autoencoder integrating RNA-seq and chromatin imaging. Differential gene expression analysis between cells with central and peripheral chromatin pattern performed on the predicted gene expression matrix translated from images using our methodology with different architecture choices. The predicted fold-change of gene expression based on images is strongly correlated with the observed fold-change of gene expression between quiescent and poised naive T-cells from the actual RNA-seq dataset. (a) Original model with 128 latent dimensions and 4 layers in the RNA-seq VAE, (b) model with 256 latent dimensions, (c) model with 3 layers in the RNA-seq VAE, (d) model with 5 layers in the RNA-seq VAE, (e) model with fully-connected image VAE instead of convolutional.

Supplementary Tables

	**Input size**	**Hidden layer size(s)**	**Output size**
**Encoder A**	815 (ATAC-seq TFs)	815, 815, 815, 100	50 (latent space)
**Encoder B**	2613 (RNA-seq genes)	2613, 2613, 2613, 100	50 (latent space)
**Decoder A**	50 (latent space)	100, 815, 815, 815	815 (ATAC-seq TFs)
**Decoder B**	50 (latent space)	100, 2613, 2613, 2613	2613 (RNA-seq genes)
**Discriminator**	50 (latent space)	50, 100	1
**Classifier**	50 (latent space)	N/A	3 (treatment time class probabilities)

Supplementary Table 1: Network architecture for autoencoder network trained on RNA-seq and ATAC-seq data collected from A549 cells. The discriminator, decoders, and encoders have leaky ReLU activations after each layer.

Loss description	Type	Weight
Reconstruction loss for ATAC-seq	Mean absolute error	10
Reconstruction loss for RNA-seq	Mean absolute error	10
Discriminative loss	Mean squared error	10
Shared cluster (treatment time) classification loss for ATAC-seq	Cross-entropy	10
Shared cluster (treatment time) classification loss for RNA-seq	Cross-entropy	10
Anchor/supervision loss between paired points in the latent space	Mean absolute error	0.1

Supplementary Table 2: Losses and corresponding weights for autoencoder network trained on RNA-seq and ATAC-seq data collected from A549 cells.

Cluster #	Differentially overexpressed genes	Cluster annotation
0	S100A8, S100A9, LYZ, S100A12, TYROBP, FCN1, FTL, CTSS, MNDA, CST3	
1	LDHB, CCR7, LEF1, RPL31, NOSIP, CD3E, RPS27, RPS6, SARAF, TCF7	Naive CD4+ T-cells
2	CCL5, NKG7, GZMK, GZMA, IL32, KLRB1, CST7, DUSP2, CMC1, CTSW	Cytotoxic T-cells
3	IL32, LTB, IL7R, ITGB1, KLRB1, LDHB, CD3D, CD2, AQP3, GSTK1	Activated CD4+ T-cells
4	CD8B, CD8A, JUNB, LDHB, LEF1, CCR7, NPM1, RPS6, CD7, SARAF	Naive CD8+ T-cells
5	TCL1A, CD79A, CD74, CD79B, MS4A1, HLA-DRA, HLA-DPA1, HLA-DQB1, HLA-DPB1, CD37	
6	CD79A, MS4A1, CD79B, CD74, JCHAIN, HLA-DRA, HLA-DPA1, HLA-DPB1, HLA-DQB1, BANK1	
7	GNLY, NKG7, PRF1, FGFBP2, GZMA, CTSW, KLRD1, GZMB, KLRF1, SPON2	
8	LST1, FCGR3A, AIF1, SAT1, FCER1G, COTL1, PSAP, MS4A7, FTL, IFITM3	
9	HLA-DQA1, HLA-DRB1, CST3, HLA-DPB1, HLA-DPA1, FCER1A, HLA-DRA, CD74, HLA-DQB1, LYZ	
10	PPBP, PF4, GNG11, HIST1H2AC, RGS18, TUBB1, TSC22D1, S100A9, S100A8, NRGN	
11	CD79A, CD79B, CD74, TCL1A, MS4A1, CD37, BANK1, HLA-DPA1, CD22, RALGPS2	
12	GZMB, JCHAIN, LILRA4, ITM2C, PTGDS, IRF7, IRF8, PLD4, PLAC8, CCDC50	

Supplementary Table 3: Top 10 differentially upregulated genes (average log-fold change > 0) for each cluster in PBMC data set.

	Image autoencoder	RNA-seq autoencoder
Encoder	2D Convolutional Block (1, 128, 4 x 4, 2)<br>2D Convolutional Block (128, 256, 4 x 4, 2)<br>2D Convolutional Block (256, 512, 4 x 4, 2)<br>2D Convolutional Block (512, 1024, 4 x 4, 2)<br>2D Convolutional Block (1024, 1024, 4 x 4, 2)<br>Fully connected (4096, 128)	Fully connected block (7633, 1024)<br>Fully connected block (1024, 1024)<br>Fully connected block (1024, 1024)<br>Fully connected block (1024, 1024)<br>Fully connected block (1024, 1024)<br>Fully connected (1024, 128)
Decoder	Fully connected (128, 4096)<br>2D Transposed Convolutional Block (1024, 1024, 4 x 4, 2)<br>2D Transposed Convolutional Block (1024, 512, 4 x 4, 2)<br>2D Transposed Convolutional Block (512, 256, 4 x 4, 2)<br>2D Transposed Convolutional Block (256, 128, 4 x 4, 2)<br>2D Transposed Convolutional Block (128, 1, 4 x 4, 2)<br>Sigmoid	Fully connected (128, 1024)<br>Fully connected block (1024, 1024)<br>Fully connected block (1024, 1024)<br>Fully connected block (1024, 1024)<br>Fully connected block (1024, 1024)<br>Fully connected (1024, 7633)

Supplementary Table 4: Network architecture for RNA-seq and image autoencoder networks. Each block consists of a batch normalization layer and ReLU nonlinearity. The discriminator has the same structure as the RNA-seq decoder with no batch normalization, 3 fully connected blocks and output dimension of 2.

Supplementary References

1. Cao, J. *et al.* Joint profiling of chromatin accessibility and gene expression in thousands of single cells. *Science* **361,** 1380–1385 (2018).
2. Andrew, G., Arora, R., Bilmes, J. & Livescu, K. *Deep canonical correlation analysis* in *Proceedings of the 30th International Conference on Machine Learning* **28** (2013), 1247–1255.
3. Butler, A., Hoffman, P., Smibert, P., Papalexi, E. & Satija, R. Integrating single-cell transcriptomic data across different conditions, technologies, and species. *Nat. Biotechnol.* **36,** 411–420 (2018).
4. Stuart, T. *et al.* Comprehensive integration of single-cell data. *Cell* **177,** 1888–1902 (2019).
5. Zhu, J.-Y., Park, T., Isola, P. & Efros, A. A. *Unpaired image-to-image translation using cycle-consistent adversarial networks* in *Proceedings of the IEEE International Conference on Computer Vision* (2017), 2223–2232.
6. Amodio, M. & Krishnaswamy, S. *MAGAN: Aligning Biological Manifolds* in *Proceedings of the 35th International Conference on Machine Learning* **80** (2018), 215–223.
7. Zheng, G. X. *et al.* Massively parallel digital transcriptional profiling of single cells. *Nat. Commun.* **8,** 14049 (2017).
8. Willinger, T. *et al.* Human naive CD8 T cells down-regulate expression of the WNT pathway transcription factors lymphoid enhancer binding factor 1 and transcription factor 7 (T cell factor-1) following antigen encounter in vitro and in vivo. *The Journal of Immunology* **176,** 1439–1446 (2006).
9. Ding, J. *et al.* Systematic comparative analysis of single cell RNA-sequencing methods. *Nat. Biotechnol.* **38,** 737–746 (2020).
