# Supplementary material for: Multi-domain translation between single-cell imaging and sequencing data using autoencoders
Source: Nat Commun. 2021 Jan 4;12:31. doi: 10.1038/s41467-020-20249-2 (PMC7782789; doi:10.1038/s41467-020-20249-2)
Supplement: Supplementary file 3 — Description of Additional Supplementary Files [file 41467_2020_20249_MOESM3_ESM.pdf]

Description of Additional Supplementary Files

File Name: Supplementary Data 1
Description: Transcription factor motif by cells matrix for ATAC-seq data from A549 cells.

File Name: Supplementary Data 2
Description: Cluster label assignments based on single-cell RNA-seq for PBMC cells. Cluster label assignment for naive CD4+ T-cells based on single-cell RNA-seq. Differential expression of genes between quiescent and poised naive CD4+ T-cells. Gene expression matrix corresponding to naive CD4+ T-cells.
