# Supplementary material for: Multi-domain translation between single-cell imaging and sequencing data using autoencoders
Source: Nat Commun. 2021 Jan 4;12:31. doi: 10.1038/s41467-020-20249-2 (PMC7782789; doi:10.1038/s41467-020-20249-2)
Supplement: Supplementary file 6 — Reporting Summary [file 41467_2020_20249_MOESM6_ESM.pdf]

nature research

Corresponding author(s): Caroline Uhler

Last updated by author(s): Nov 6, 2020

Reporting Summary

Nature Research wishes to improve the reproducibility of the work that we publish. This form provides structure for consistency and transparency in reporting. For further information on Nature Research policies, see Authors & Referees and the Editorial Policy Checklist.

Statistics

For all statistical analyses, confirm that the following items are present in the figure legend, table legend, main text, or Methods section.

n/a	Confirmed	
☐	☒	The exact sample size (*n*) for each experimental group/condition, given as a discrete number and unit of measurement
☒	☐	A statement on whether measurements were taken from distinct samples or whether the same sample was measured repeatedly
☐	☒	The statistical test(s) used AND whether they are one- or two-sided<br>*Only common tests should be described solely by name; describe more complex techniques in the Methods section.*
☒	☐	A description of all covariates tested
☐	☒	A description of any assumptions or corrections, such as tests of normality and adjustment for multiple comparisons
☐	☒	A full description of the statistical parameters including central tendency (e.g. means) or other basic estimates (e.g. regression coefficient) AND variation (e.g. standard deviation) or associated estimates of uncertainty (e.g. confidence intervals)
☐	☒	For null hypothesis testing, the test statistic (e.g. *F*, *t*, *r*) with confidence intervals, effect sizes, degrees of freedom and *P* value noted<br>*Give P values as exact values whenever suitable.*
☒	☐	For Bayesian analysis, information on the choice of priors and Markov chain Monte Carlo settings
☒	☐	For hierarchical and complex designs, identification of the appropriate level for tests and full reporting of outcomes
☐	☒	Estimates of effect sizes (e.g. Cohen's *d*, Pearson's *r*), indicating how they were calculated

Our web collection on statistics for biologists contains articles on many of the points above.

Software and code

Policy information about availability of computer code

Data collection	No software was used for data collection.
Data analysis	PyTorch (1.2.0), Seurat (2.3.0, 3.0), g:Profiler (e97_eg44_p13_2fcb244), scikit-learn (0.21.3), scipy (1.3.1), numpy(1.17.2), seaborn(0.9.0), Fiji (1.52), R version (3.4.3), https://github.com/junyanz/pytorch-CycleGAN-and-pix2pix, https://github.com/uhlerlab/cross-modal-autoencoders, https://github.com/SaradhaVenkatachalapathy/Radial_chromatin_packing_immune_cells

For manuscripts utilizing custom algorithms or software that are central to the research but not yet described in published literature, software must be made available to editors/reviewers. We strongly encourage code deposition in a community repository (e.g. GitHub). See the Nature Research guidelines for submitting code & software for further information.

Data

Policy information about availability of data

All manuscripts must include a data availability statement. This statement should provide the following information, where applicable:

- Accession codes, unique identifiers, or web links for publicly available datasets
- A list of figures that have associated raw data
- A description of any restrictions on data availability

The data for model validation on paired single-cell RNA-seq and ATAC-seq is publicly available and was obtained from GSE117089. The RNA-seq data for integration of RNA-seq and chromatin images is publicly available and was obtained from https://support.10xgenomics.com/single-cell-gene-expression/datasets/2.1.0/pbmc8k. The chromatin images are available at Zenodo from DOI: 10.5281/zenodo.4265737.

Field-specific reporting

Please select the one below that is the best fit for your research. If you are not sure, read the appropriate sections before making your selection.

☒ Life sciences ☐ Behavioural & social sciences ☐ Ecological, evolutionary & environmental sciences

For a reference copy of the document with all sections, see nature.com/documents/nr-reporting-summary-flat.pdf

Life sciences study design

All studies must disclose on these points even when the disclosure is negative.

Sample size	A minimum of 200 cells were imaged in each experiment. In our previous work on t-cell activation, we used a sample size of 150 per experiment (https://doi.org/10.1371/journal.pone.0043718). Hence, in this work sample size of at least 200 cells per experiment was used to capture the variability in the samples.
Data exclusions	Cells that were not fully imaged or were saturated were excluded. These exclusion criteria were applied to the images acquired using the confocal microscope.
Replication	All experiments were successfully replicated at least 3 times.
Randomization	We imaged random cells in the imaging dish and complied the data from across multiple experiments. Randomization of samples doesnt apply here as we had only one sample.
Blinding	Blinding was applied to the data analysis.

Reporting for specific materials, systems and methods

We require information from authors about some types of materials, experimental systems and methods used in many studies. Here, indicate whether each material, system or method listed is relevant to your study. If you are not sure if a list item applies to your research, read the appropriate section before selecting a response.

Materials & experimental systems

n/a	Involved in the study	
☐	☒	Antibodies
☐	☒	Eukaryotic cell lines
☒	☐	Palaeontology
☒	☐	Animals and other organisms
☒	☐	Human research participants
☒	☐	Clinical data

Methods

n/a	Involved in the study	
☒	☐	ChIP-seq
☒	☐	Flow cytometry
☒	☐	MRI-based neuroimaging

Antibodies

Antibodies used	The primary antibodies used in this study are anti-RPL10A antibody (Abcam, ab174318, dilution 1/200) and Anti-Coronin 1a/TACO antibody (Abcam ab14787, dilution 1/150).
Validation	Both antibodies were validated using western blot by the manufacturer. Anti-RPL10A antibody [EPR12344] (Abcam ab174318) is a Rabbit monoclonal antibody . It is expected to react with Mouse, Rat and Human samples. Manufacturer validated the antibody by performing Western blot for cell lysates from Jurkat, HeLa, HepG2 and PC-12, which resulted in a band at the predicted molecular weight of 25kDa. Furthermore, immunohistochemical analysis of paraffin-embedded human endometrial adenocarcinoma tissue and immunofluorescence analysis of HeLa cells was performed. Anti-Coronin 1a/TACO antibody (Abcam ab14787) is a Goat polyclonal antibody. It is expected to react with Mouse and Human samples. Manufacturer validated the antibody by performing Western blot for cell lysates from Jurkat and MOLT4, HepG2 and PC-12, which resulted in a band at the predicted molecular weight of 60kDa. In transfected HEK293 cells a band of ~50kDa is observed.

Eukaryotic cell lines

Policy information about cell lines

Cell line source(s)	CD4+/CD45RA+ naive helper T cells from human peripheral blood (custom order from AllCells)
Authentication	Cells were authenticated by the manufacturer. We used CD4+/CD45RA+ naive helper T cells from human peripheral blood. The manufacturer (AllCells) characterized the cells isolated from donors by immunophenotyping (by flow cytometry) for surface markers.

Mycoplasma contamination	Cells were not tested for Mycoplasma contamination
Commonly misidentified lines<br>(See ICLAC register)	No commonly misidentified cell lines were used.
